# Supplementary material for: An electricity-driven mobility circular economy with lifecycle carbon footprints for climate-adaptive carbon neutrality transformation
Source: Nat Commun. 2024 Jul 13;15:5905. doi: 10.1038/s41467-024-49868-9 (PMC11246450; doi:10.1038/s41467-024-49868-9)
Supplement: Supplementary file 1 — Supplementary Information [file 41467_2024_49868_MOESM1_ESM.pdf]

Supplementary information for

An electricity-driven mobility circular economy with lifecycle carbon footprints for climate-adaptive carbon neutrality transformation

Aoye Song[1,2 #], Zhaohui Dan[1 #], Siqian Zheng[1,3*] & Yuekuan Zhou[1,2,4,5*]

[1]Sustainable Energy and Environment Thrust, Function Hub, The Hong Kong University of Science and Technology (Guangzhou), Nansha, Guangzhou, 511400, Guangdong, China

[2]Division of Emerging Interdisciplinary Areas, The Hong Kong University of Science and Technology, Clear Water Bay, Hong Kong SAR, China

[3]Hong Kong Productivity Council, Tat Chee Ave, Kowloon, Hong Kong SAR, China

[4]Department of Mechanical and Aerospace Engineering, The Hong Kong University of Science and Technology, Clear Water Bay, Hong Kong SAR, China

[5]HKUST Shenzhen-Hong Kong Collaborative Innovation Research Institute, Futian, Shenzhen 518048, China

[#]These authors contributed equally: Aoye Song, Zhaohui Dan

*Corresponding author:

Email: yuekuanzhou@hkust-gz.edu.cn (Zhou Y.)

This file includes:

Supplementary Tables 1 to 26

Supplementary Figures 1 to 13

Supplementary Notes 1 to 9

Supplementary Table 1. The carbon intensity of different scenarios in Guangzhou.

Scenarios	A	B	C	D	E
Carbon intensity (kg $CO_{2,e}$ kWh^{-1})	1,625.01	-1,104.91	108.04	504.06	-904.41

Supplementary Table 2. Polynomial coefficients (*k*) of battery degradation model in different depth of discharge (*DoD*).

	DoD = 1	*DoD* = 0.8	*DoD* = 0.6	*DoD* = 0.4	*DoD* = 0.3	*DoD* = 0.2	*DoD* = 0.1
k_1	-2.685×10^{-11}	-8.732×10^{-12}	-2.562×10^{-12}	-5.362×10^{-13}	-3.084×10^{-13}	-1.934×10^{-13}	-1.292×10^{-13}
k_2	1.539×10^{-7}	6.271×10^{-8}	2.665×10^{-8}	9.537×10^{-9}	6.622×10^{-9}	4.866×10^{-9}	3.727×10^{-9}
k_3	-3.261×10^{-4}	-1.947×10^{-4}	-1.276×10^{-4}	-7.764×10^{-5}	-6.492×10^{-5}	-5.581×10^{-5}	-4.894×10^{-5}
k_4	1	1	1	1	1	1	1

Supplementary Table 3. The electrical performance of the installed photovoltaic module.

Electrical performance parameters (STC[a])			
Type	Hiku-CS3W-450 MS[1]	Operation temperature	-40~85 °C
Maximum power (P_{max})	450 W	Size	2,108×1,048×35 mm
Voltage at maximum power (V_{mp})	41.1 V	Temperature coefficient (Pmax)	-0.34% / °C
Current at maximum power (I_{mp})	10.96 A	Temperature coefficient (Pmax)	-0.26% / °C
Open-circuit voltage (V_{oc})	49.1	Temperature coefficient (Pmax)	0.05% / °C
Short-circuit current (I_{sc})	11.60 A	Normal Operating Cell Temperature	41±3 °C
Efficiency	20.39%		

[a] Standard testing conditions(STC): Solar radiation at 1,000 W m^{-2}, Cell temperature at 25 °C

Supplementary Table 4. The installed power of wind turbines in the Net-zero energy paradigm.

Cities	Buildings	500 kW Wind turbine	150 kW Wind turbine	20 kW Wind turbine	Total installed power / kW
Xi'an	Hotel	11			10,300
	Office	6			
	Residence			90	
Shenzhen	Hotel	2			1,720
	Office		2		
	Residence			21	
Hong Kong	Hotel	1			950
	Office		1		
	Residence			15	
Guangzhou	Hotel	9			7,200
	Office	3			
	Residence			60	
Beijing	Hotel	6			6,200
	Office	4			
	Residence			60	
Shanghai	Hotel	4			3,720
	Office	2			
	Residence			36	
Kunming	Hotel	8			5,280
	Office	1			
	Residence			39	
New York	Hotel		4		1,200
	Office		2		
	Residence			15	
Berlin	Hotel	2			2,140

	Office		4		
	Residence			27	
	Hotel	7			
Singapore	Office	2			5,700
	Residence			60	

Supplementary Table 5. The installed power of wind turbines in low-positive energy paradigm.

Cities	Buildings	500 kW Wind turbine	150 kW Wind turbine	20 kW Wind turbine	Total installed power / kW
Xi'an	Hotel	19			18,800
	Office	12			
	Residence			165	
Shenzhen	Hotel	3			3,030
	Office		5		
	Residence			39	
Hong Kong	Hotel		6		1,830
	Office		3		
	Residence			24	
Guangzhou	Hotel	16			13,720
	Office	7			
	Residence			111	
Beijing	Hotel	10			10,600
	Office	7			
	Residence			105	
Shanghai	Hotel	7			7,260
	Office	5			
	Residence			63	
Kunming	Hotel	16			12,680
	Office	6			
	Residence			84	

Supplementary Table 6 The installed power of wind turbines in a high-positive energy paradigm.

Cities	Buildings	500 kW Wind turbine	150 kW Wind turbine	20 kW Wind turbine	Total installed power / kW
Xi'an	Hotel	27			27,800
	Office	19			
	Residence			240	
Shenzhen	Hotel	5			4,840
	Office		8		
	Residence			57	
Hong Kong	Hotel		8		2,520
	Office		4		
	Residence			36	
Guangzhou	Hotel	23			20,800
	Office	12			
	Residence			165	
Beijing	Hotel	14			15,000
	Office	10			
	Residence			150	
Shanghai	Hotel	11			10,800
	Office	7			
	Residence			90	
Kunming	Hotel	23			19,520
	Office	11			
	Residence			126	

Supplementary Table 7 Parameters of wind turbines[2, 3, 4].

Parameters	Information and Value		
Wind turbine	AN Bonus 150/30	Vestas V39	Hummer-h13.2-20kW
Rated power (kW)	150	500	20
Cut-in wind speed ($m{\cdot}s^{-1}$)	4	5	3
Rated speed ($m{\cdot}s^{-1}$)	12.5	15	9
Cut-out wind speed ($m{\cdot}s^{-1}$)	25	25	25
Hub height (m)	40	53	19.4
Rotor diameter (m)	23	39	13.2

Supplementary Table 8 Monthly bulk tariff in Hong Kong.

$P_{peak,max}$ (kVA)	$C_{peak,max}$[a] (HK$ kVA^{-1})[d]	$P_{off\text{-}peak,max}$ (kVA)	$C_{off\text{-}peak,max}$[b] (HK$ kVA^{-1})	$E_{eg,imp}$ (kWh)	$C_{eg,imp}$[c] (HK$ kWh^{-1})	
	Peak[d]		Off-peak[e]		Peak[d]	Off-peak[e]
≤ 650 kVA	68.4	≤ $P_{peak,max}$	0	≤ 200,000	0.753	0.676
> 650 kVA	65.4	> $P_{peak,max}$	26.8	> 200,000	0.737	0.676

[a] $C_{peak,max}$ is the demand charge price during the peak period.

[b] $C_{off\text{-}peak,max}$ is the demand charge price during the off-peak period.

[c] $C_{eg,imp}$ is the energy charge price.

[d] 1 HK$=0.1274 US$

[e] Peak period is from 9:00 AM to 21:00 PM.

[f] Off-peak period is from 21:00 PM to 0:00 AM and from 0:00 AM to 9:00 AM.

Supplementary Table 9 Residential tariff in Hong Kong.

E_{imp} (every two months per household, kWh)	≤400	>400 and ≤1,000	>1,000 and ≤1,800	>1,800 and ≤2,600	>2,600 and ≤3,400	> 3,400 and ≤4,200	>4,200
$C_{eg,imp}$ (HK$ kWh^{-1})[a]	0.870	1.004	1.162	1.470	1.699	1.803	1.815

[a] 1 HK$=0.1274 US$

Supplementary Table 10 Electricity price for residential buildings in Beijing.

E_{imp} (each month per household, kWh)	≤ 240	> 240 and ≤ 400	> 400
$C_{eg,imp,r}$ (CN¥ kWh^{-1})[a]	0.4883	0.5383	0.7883

[a] 1 CN¥=0.1429 US$

Supplementary Table 11 Electricity price for commercial users in Beijing.

Period	Super peak[a]	Peak[b]	Flat[c]	Valley[d]
$C_{eg,imp,c}$ (CN¥ kWh^{-1})[e]	1.2713	1.1574	0.8617	0.5953

[a] Super peak period is from 11:00 AM to 13:00 PM and from 16:00 PM to 17:00 PM in July and August.

[b] Peak period is from 10:00 AM to 11:00 AM, from 13:00 PM to 15:00 PM and from 18:00 PM to 21:00 PM in July and August. In other periods (except for July and August), the peak period is from 10:00 AM to 15:00 PM and from 18:00 PM to 21:00 PM.

[c] Flat period is from 7:00 AM to 10:00 AM, from 15:00 PM to 18:00 PM and from 21:00 PM to 23:00 PM.

[d] Valley period is from 23:00 PM to 7:00 AM.

[e] 1 CN¥=0.1429 US$

Supplementary Table 12 Electricity price for residential buildings in Guangzhou.

$C_{eg,imp1}$[a] (CN¥ kWh^{-1})[b] (from May to October)				$C_{eg,imp2}$[c] (CN¥ kWh^{-1}) (from November to April)			
E_{imp} (each month per household, kWh)	Peak[d]	Flat[e]	Valley[f]	E_{imp} (each month per household, kWh)	Peak[d]	Flat[e]	Valley[f]
≤ 260	0.9950	0.5889	0.2292	≤ 200	0.9950	0.5889	0.2292
> 260 and ≤ 600	1.0450	0.6389	0.2792	> 200 and ≤ 400	1.0450	0.6389	0.2792
> 600	1.2950	0.8889	0.5292	> 400	1.2950	0.8889	0.5292

[a] $C_{eg,imp1}$ is the import cost from May to October (summer period).

[b] 1 CN¥=0.1429 US$

[c] $C_{eg,imp2}$ is the import cost from November to April (non-summer period).

[d] Peak period is from 10:00 AM to 12:00 PM and from 14:00 PM to 19:00 PM.

[e] Flat period is from 8:00 AM to 10:00 AM, from 12:00 PM to 14:00 PM and from 19:00 PM to 24:00 PM.

[f] Valley period is from 0:00 AM to 8:00 AM.

Supplementary Table 13 Electricity price for commercial users in Guangzhou.

Period	Super peak[a]	Peak[b]	Flat[c]	Valley[d]
$C_{eg,imp,c}$ (CN¥ kWh^{-1})[e]	1.7156	1.3780	0.8220	0.3296

[a] Super peak period is from 11:00 AM to 12:00 PM and from 15:00 PM to 17:00 PM in July, August, and September.

[b] Peak period is from 10:00 AM to 11:00 AM, from 14:00 PM to 15:00 PM and from 17:00 PM to 19:00 PM in July, August, and September. In other periods (except for July, August, and September), the peak period is from 10:00 AM to 12:00 AM and from 14:00 PM to 19:00 PM.

[c] Flat period is from 8:00 AM to 10:00 AM, from 12:00 PM to 14:00 PM and from 19:00 PM to 24:00 PM.

[d] Valley period is from 0:00 AM to 8:00 AM.

[e] 1 CN¥=0.1429 US$

Supplementary Table 14 Electricity price for residential buildings in Shenzhen.

$C_{eg,imp1}$[a] (CN¥kWh^{-1})[b] (from May to October)				$C_{eg,imp2}$[c] (CN¥ kWh^{-1}) (from November to April)			
E_{imp} (each month per household, kWh)	Peak[d]	Flat[e]	Valley[f]	E_{imp} (each month per household, kWh)	Peak[d]	Flat[e]	Valley[f]
≤ 260	1.1208	0.6629	0.2573	≤ 200	1.1208	0.6629	0.2573
> 260 and ≤ 600	1.1708	0.7129	0.3073	> 200 and ≤ 400	1.1708	0.7129	0.3073
> 600	1.4208	0.9629	0.5573	> 400	1.4208	0.9629	0.5573

[a] $C_{eg,imp1}$ is the import cost from May to October (summer period).

[b] 1 CN¥=0.1429 US$

[c] $C_{eg,imp2}$ is the import cost from November to April (non-summer period).

[d] Peak period is from 10:00 AM to 12:00 PM and from 14:00 PM to 19:00 PM.

[e] Flat period is from 8:00 AM to 10:00 AM, from 12:00 PM to 14:00 PM and from 19:00 PM to 24:00 PM.

[f] Valley period is from 0:00 AM to 8:00 AM.

Supplementary Table 15 Electricity price for commercial users in Shenzhen.

Period	Super peak[a]	Peak[b]	Flat[c]	Valley[d]
$C_{eg,imp,c}$ (CN¥ kWh^{-1})[e]	1.7156	1.3780	0.8220	0.3296

[a] Super peak period is from 11:00 AM to 12:00 PM and from 15:00 PM to 17:00 PM in July, August, and September.

[b] Peak period is from 10:00 AM to 12:00 AM and from 14:00 PM to 19:00 PM except for super peak period.

[c] Flat period is from 8:00 AM to 10:00 AM, from 12:00 PM to 14:00 PM and from 19:00 PM to 24:00 PM except for super peak period.

[d] Valley period is from 0:00 AM to 8:00 AM.

[e] 1 CN¥=0.1429 US$

Supplementary Table 16 Electricity price for residential users in Xi'an.

Period	Peak[a]	Flat[b]	Valley[c]
$C_{eg,imp,c}$ (CN¥ kWh^{-1})[d]	0.5483	0.4983	0.2983

[a] Peak period is from 8:00 AM to 11:30 AM and from 18:30 PM to 23:00 PM.

[b] Flat period is from 7:00 AM to 8:00 AM, from 11:30 AM to 18:30 PM.

[c] Valley period is from 23:00 PM to 7:00 AM.

[d] 1 CN¥=0.1429 US$

Supplementary Table 17 Electricity price for commercial users in Xi'an.

Period	Peak[a]	Flat[b]	Valley[c]
$C_{eg,imp}$ (CN¥ kWh^{-1})[d]	0.8955	0.6124	0.3294

[a] Peak period is from 8:00 AM to 11:30 AM and from 18:30 PM to 23:00 PM.

[b] Flat period is from 7:00 AM to 8:00 AM, from 11:30 AM to 18:30 PM.

[c] Valley period is from 23:00 PM to 7:00 AM.

[d] 1 CN¥=0.1429 US$

Supplementary Table 18 Electricity price for residential buildings in Shanghai.

	$C_{eg,imp}$ (CN¥ kWh^{-1})[a]	
E_{imp} (kWh per·household)	Peak[b]	Valley[c]
≤ 3,120	0.617	0.307
> 3,120 and ≤ 4,800	0.677	0.337
> 4,800	0.977	0.487

[a] 1 CN¥=0.1429 US$

[b] Peak period is from 6:00 AM to 22:00 PM.

[c] Valley period is from 22:00 PM to 6:00 AM (next day).

Supplementary Table 19 Electricity price for commercial users in Shanghai.

Period	Peak[a]	Valley[b]
$C_{eg,imp,c}$ (CN¥kWh^{-1})[c]	0.9753	0.4739

[a] Peak period is from 6:00 AM to 22:00 PM.

[b] Valley period is from 22:00 PM to 6:00 AM (next day).

[c] 1 CN¥=0.1429 US$

Supplementary Table 20 Electricity price for residential buildings in Kunming.

E_{imp} (kWh per·household)	$C_{eg,imp}$ (CN¥ kWh^{-1})[a]
≤ 1,560	0.3336
> 1,560 and ≤ 3,600	0.4236
> 3,600 and ≤ 4,680	0.4736
> 4,680	0.7736

[a] 1 CN¥=0.1429 US$

Supplementary Table 21 Electricity price for commercial users in Kunming.

Period	Peak[a]	Flat[b]	Valley[c]
$C_{eg,imp}$ (CN¥ kWh^{-1})[d]	0.6998	0.4878	0.2758

[a] Peak period is from 9:00 AM to 12:00 PM and from 18:00 PM to 23:00 PM.

[b] Flat period is from 7:00 AM to 9:00 AM, from 12:00 PM to 18:00 PM.

[c] Valley period is from 23:00 PM to 0:00 AM and from 0:00 AM to 7:00 AM.

[d] 1 CN¥=0.1429 US$

Supplementary Table 22 Electricity price for commercial users in Singapore.

Period	Peak[a]	Valley[b]
$C_{eg,imp}$ (US$ kWh^{-1})	0.21855	0.11325

[a] Peak period is from 7:00 AM to 22:00 PM

[b] Valley period is from 22:00 PM to 0:00 AM and from 0:00 AM to 7:00 AM.

Supplementary Table 23 Parameters of electric vehicles (EVs)[5, 6].

Parameters of private cars	Information and value	
Models	Tesla Y	Yutong E8
Battery capacity (kWh per car)	75	121.13
Running distance (km per day)	97.14	80.95
Electricity consumption (kWh per km)	0.118	0.417
Quantity of EVs	4	2
The maximum charge and discharge power per vehicle (kW)	10[7]	10[7]

Supplementary Table 24 Parameters of internal combustion engine vehicles (ICEVs)[8, 9].

Parameters of private cars	Information and value	
Models	Honda Accord 2023	Yutong C8
Running distance (km per day)	97.14	80.95
Fuel consumption (L km^{-1})	0.074	0.16
Quantity of ICEVs	4	2

Supplementary Table 25. Embodied carbon emission of vehicles during production and recycling phases.

Embodied carbon emission (kg $CO_{2,e}$)	Electric cars	Internal combustion engine cars	Electric buses	Internal combustion engine buses
Production (Vehicle)	10,952	11,235	66,928	73,028
Production (Battery)	6,375	N/A	10,296	
Recycling (Vehicle)	1,912	1,904	11,683	12,376
Recycling (Battery)	5,235	N/A	8,455	

Supplementary Table 26. Carbon emission of vehicles during the operation phase.

Operation carbon emission (kg $CO_{2,e}$)	Electric cars	Internal combustion engine cars	Electric buses	Internal combustion engine buses
Beijing	39,489.61		137,955.65	
Guangzhou	34,067.61		119,014.09	
Hong Kong	38,119.34		133,168.67	
Kunming	34,067.61	45,361.80	119,014.09	130,992.00
Shanghai	33,591.17		117,349.65	
Shenzhen	34,067.61		119,014.09	
Xi’an	37,532.65		131,119.08	

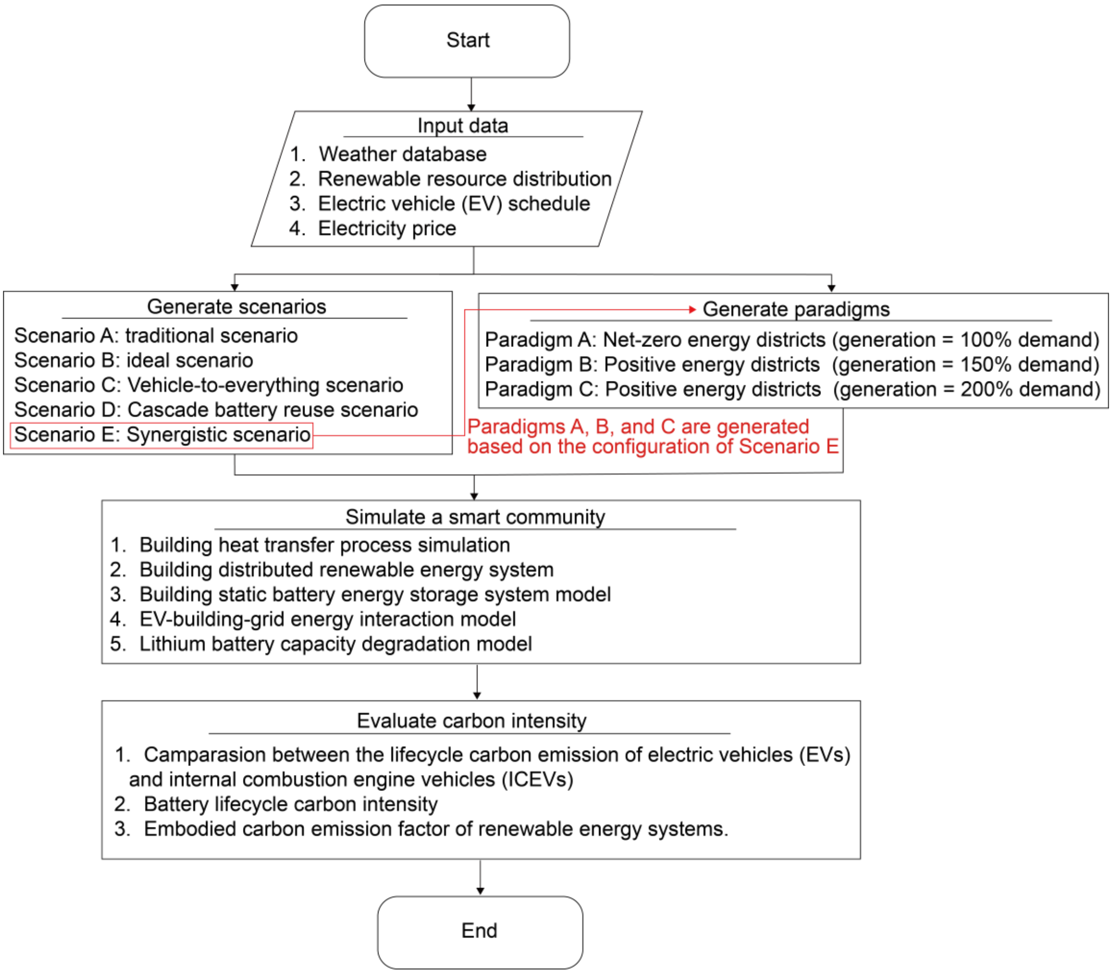

Supplementary Figure 1 The methodology flow chart of this study. The research process of this study is divided into four steps, data input, generation of energy scenarios and paradigms, simulation of smart communities and evaluation of carbon intensity.

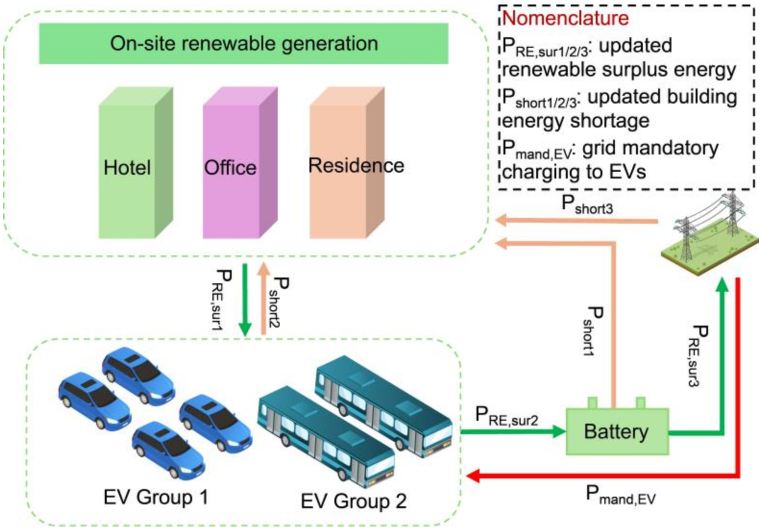

Supplementary Figure 2 The operating principle of building-transportation energy sharing network between buildings and electric vehicles (EVs). Renewable energy generation follows this order of energy flow: priority is given to meeting the energy demands of buildings, with any excess energy being used to charge electric vehicles, followed by charging energy storage batteries, and finally, exporting to the grid. Energy for meeting building energy consumption follows this pattern: priority is given to meeting the energy demands of buildings using renewable energy sources, with any shortage being supplemented by energy from storage batteries, electric vehicles, and the grid. The energy consumption demands of electric vehicles are met using excess renewable energy and energy from the grid.

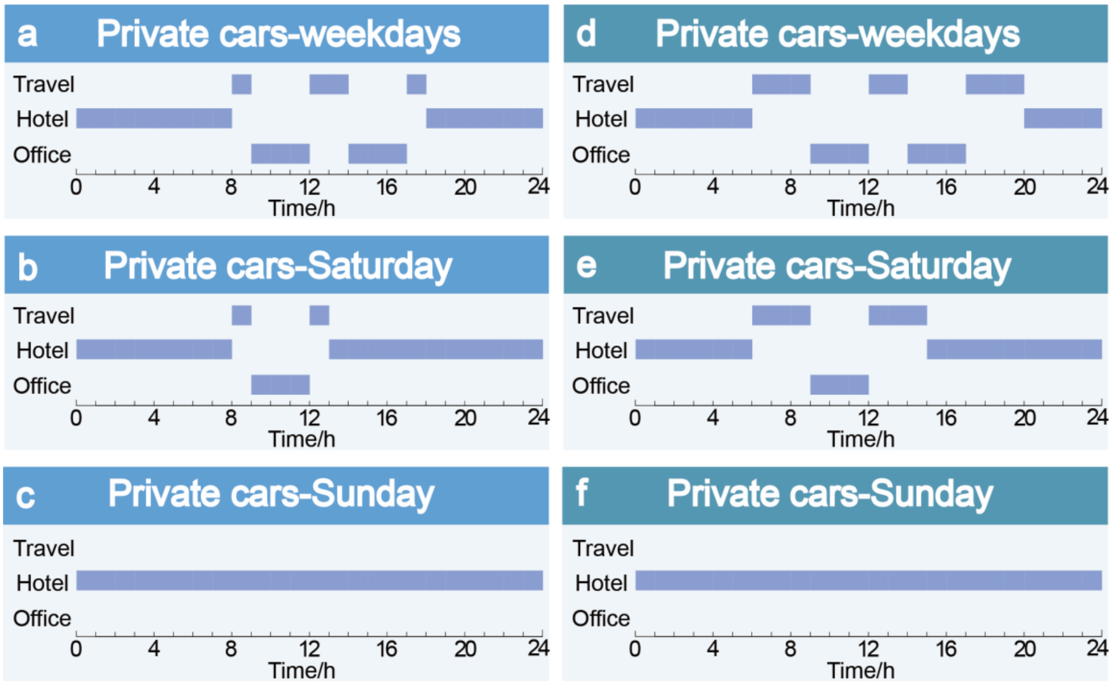

Supplementary Figure 3 The travelling schedule of private cars. a, b, c The Schedule A of private cars on weekdays, Saturday and Sunday, respectively; **d, e, f** The Schedule C of private cars on weekdays, Saturday and Sunday, respectively.

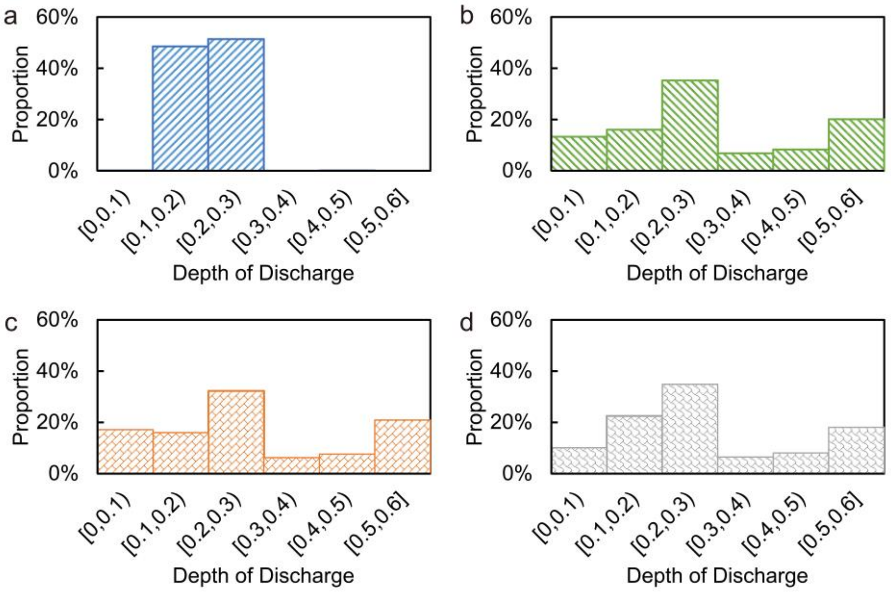

Supplementary Figure 4 The depth of discharge distribution of different schedules. a No Vehicle-to-everything (V2X); **b** Schedule A; **c** Schedule B; **d** Schedule C.

(Note: No V2X refer to the situation that there is no Vehicle-to-everything (V2X) interaction. Schedule A means that cars will run for 4 hours a day, stop at the office for 8 hours, and stop at the hotel or residential building for 12 hours. Schedule B means that cars will run for 6 hours a day, stop at the office for 8 hours, and stop at the hotel or residential building for 10 hours. Schedule C means that cars will run for 8 hours a day, stop at the office for 8 hours, and stop at the hotel or residential building for 8 hours.)

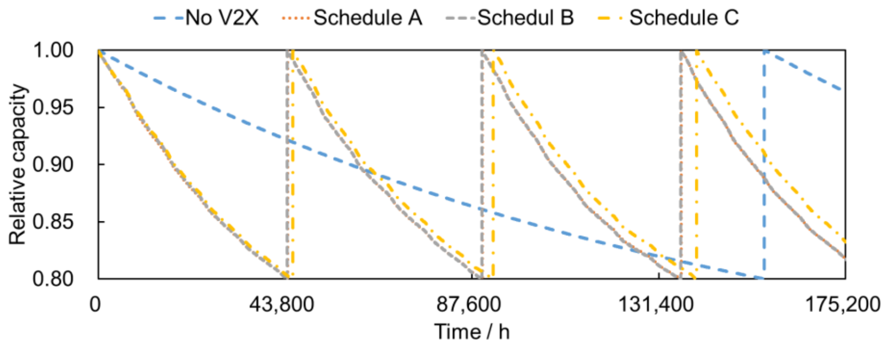

Supplementary Figure 5 The evolution on degradation of different schedules.

(Note: No V2X refer to the situation that there is no Vehicle-to-everything (V2X) interaction. Schedule A means that cars will run for 4 hours a day, stop at the office for 8 hours, and stop at the hotel or residential building for 12 hours. Schedule B means that cars will run for 6 hours a day, stop at the office for 8 hours, and stop at the hotel or residential building for 10 hours. Schedule C means that cars will run for 8 hours a day, stop at the office for 8 hours, and stop at the hotel or residential building for 8 hours.)

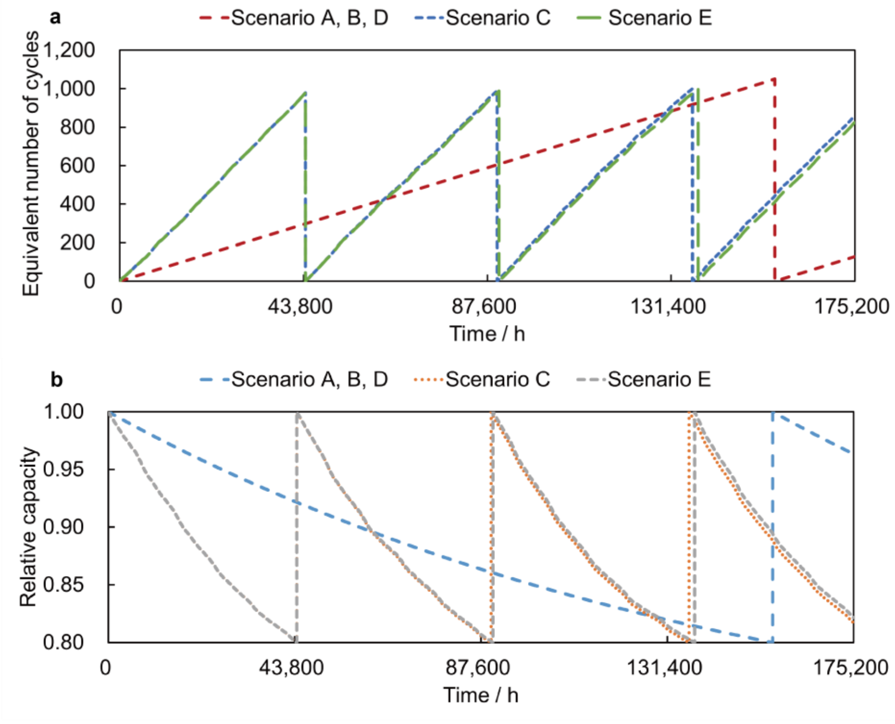

Supplementary Figure 6 The Equivalent number of cycles and evolution of relative capacity. a Equivalent number of cycles of private EVs in Guangzhou under different scenarios, **b** The evolution of relative capacity of private EV batteries under different scenarios.

(Note: Scenario A, B, D without multi-direction vehicle-to-everything (V2X) interaction, Scenario C and E with V2X interaction. The equivalent number of cycles is reset around 1000 because a new battery is replaced.)

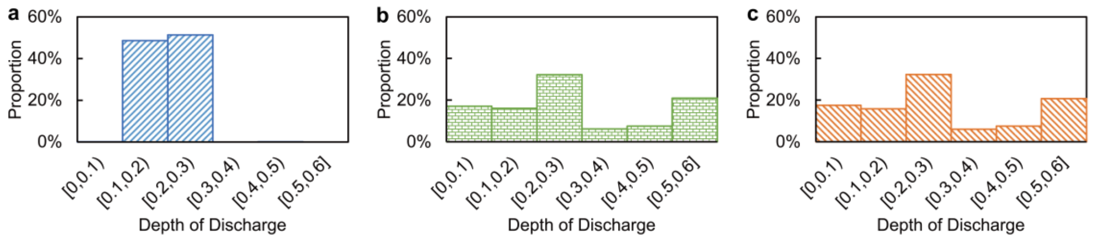

Supplementary Figure 7 Depth of discharge (DoD) distribution of private electric vehicle batteries in different scenarios. a Scenario A, B, D; **b** Scenario C; **c** Scenario E.

(Note: Scenario A, B, D without multi-direction vehicle-to-everything (V2X) interaction, Scenario C and E with V2X interaction.)

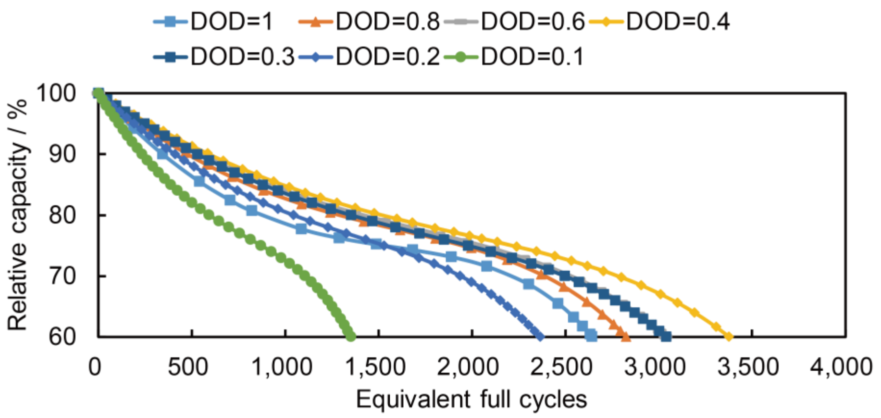

Supplementary Figure 8 Battery degradation curves based on the depth of discharge (DOD) and equivalent full cycles. This battery degradation curve shows the relative capacity remaining after the battery has undergone different equivalent full cycles. Equivalent full cycles refer to the complete charging and discharging of a battery with its initial capacity. Relative capacity refers to the ratio of the current capacity to its initial capacity of a battery.

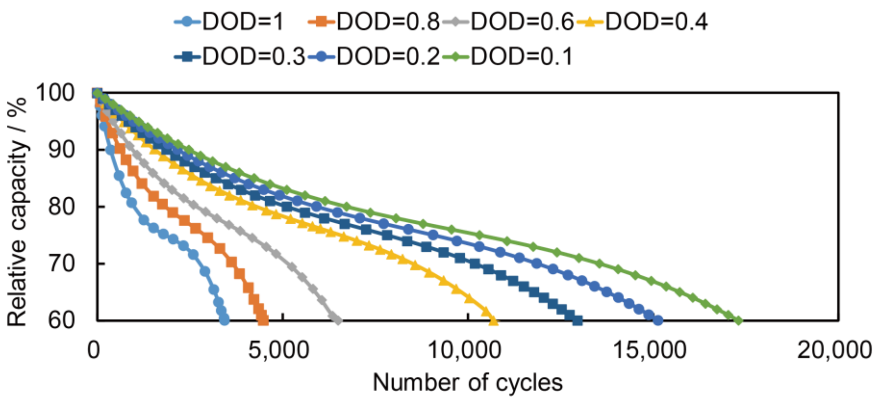

Supplementary Figure 9 Battery degradation curves based on the depth of discharge (DOD) and number of cycles. This battery degradation curve shows the relative capacity remaining after the battery has undergone different number of cycles. Number of cycles indicate the number of times that the battery has been charged and discharged. Relative capacity refers to the ratio of the current capacity to its initial capacity of a battery.

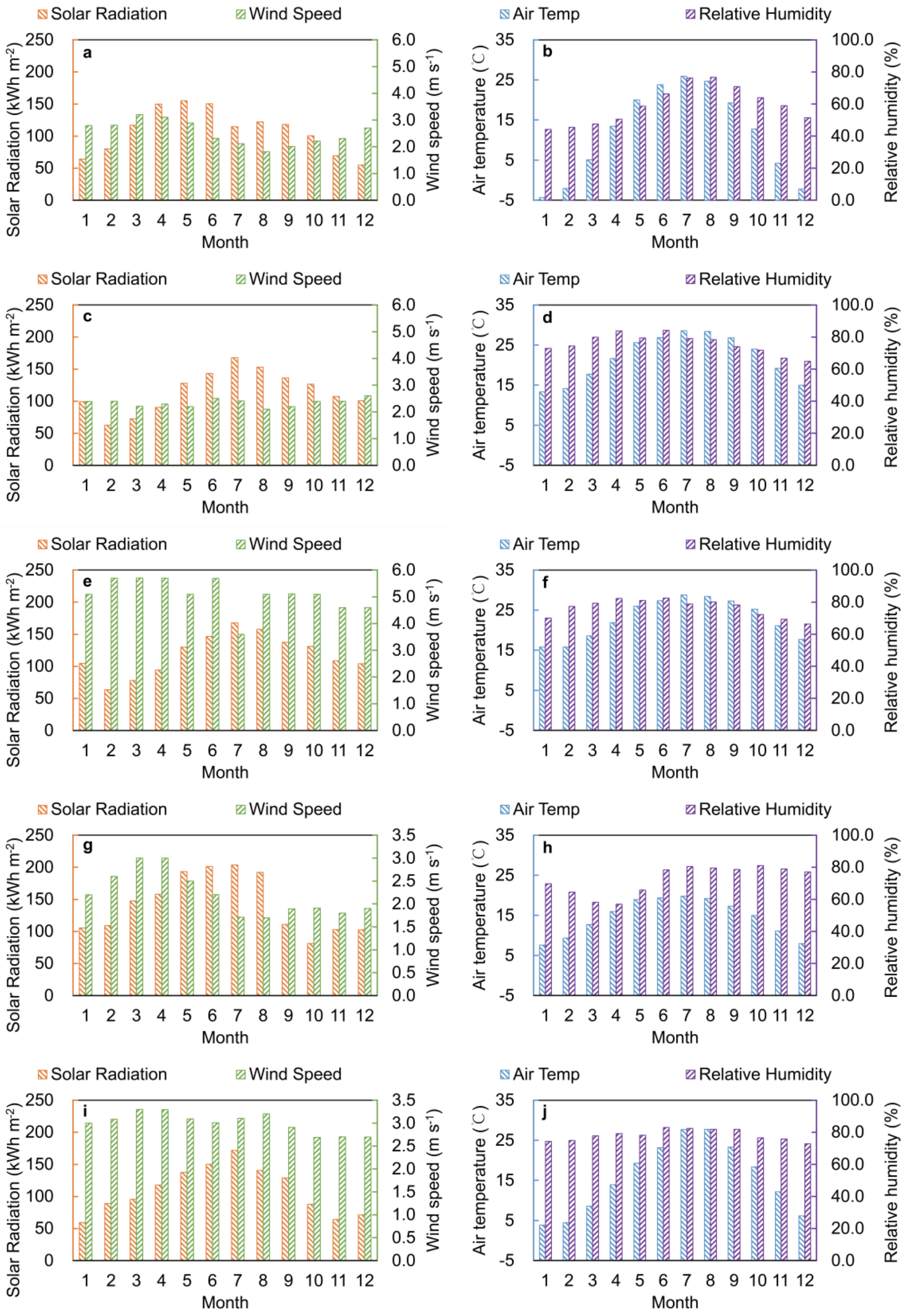

Supplementary Figure 10 The climate information of different regions. The monthly solar radiation and monthly average wind speed in Beijing (**a**), Guangzhou (**c**), Hong Kong (**e**), Kunming (**g**), Shanghai (**i**), Shenzhen (**k**), Xi'an (**m**), New York (**o**), Berlin (**q**) and Singapore (**s**); The monthly average air temperature (Air Temp) and relative humidity in Beijing (**b**), Guangzhou

(**d**), Hong Kong (**f**), Kunming (**h**), Shanghai (**j**), Shenzhen (**l**), Xi'an (**n**), New York (**p**), Berlin (**r**) and Singapore (**t**).

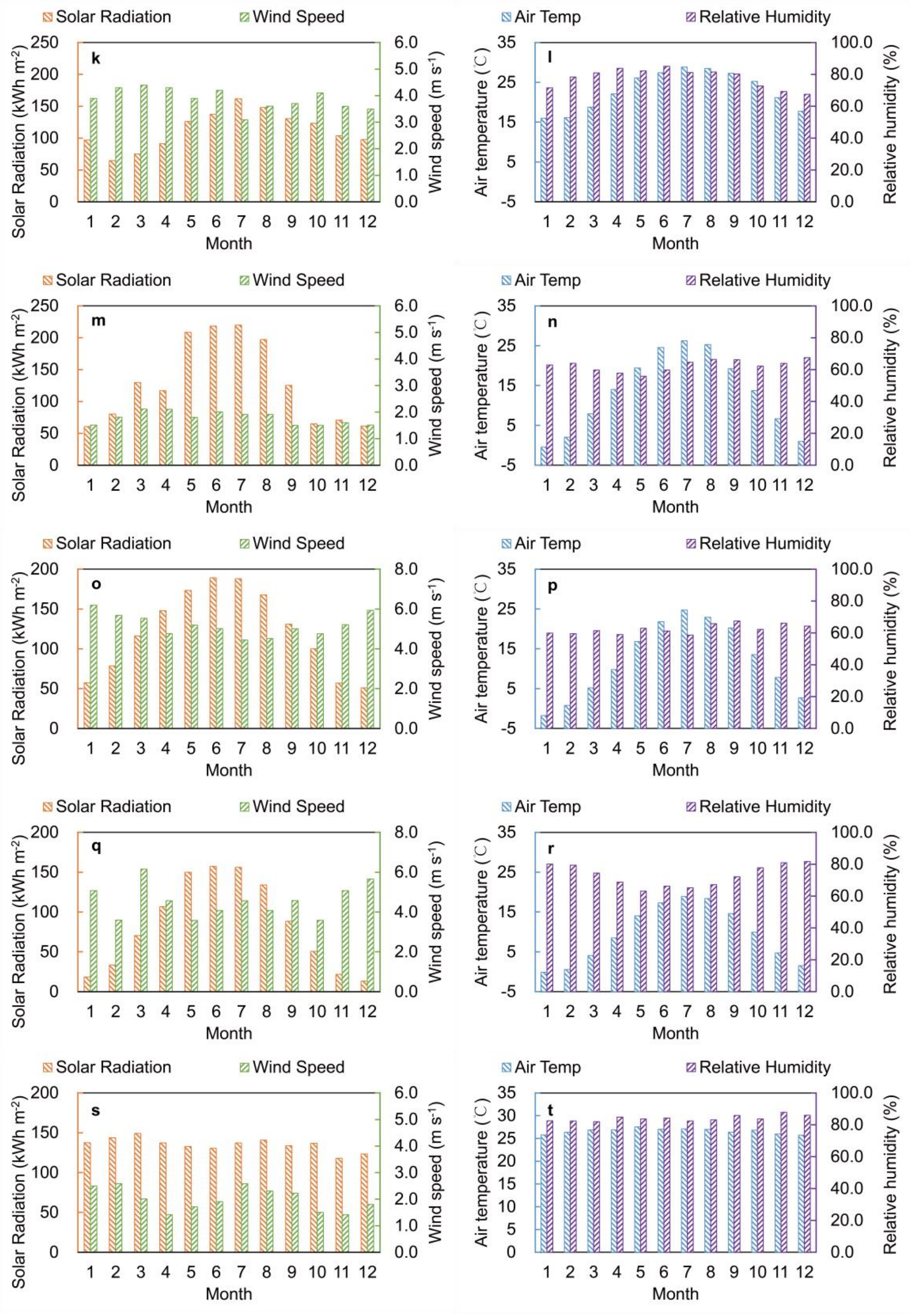

Supplementary Figure 10 (*continued*)

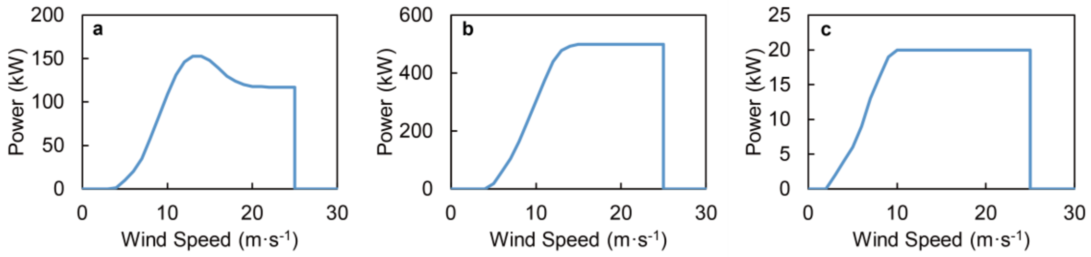

Supplementary Figure 11 The power-wind speed curve of wind turbines. a AN Bonus 150-150 kW[2]; **b** Vestas-V39-500 kW[3]; **c** Hummer-H13.2-20 kW[4]. The power-wind speed curve indicates the power generated by the wind turbine at different wind speeds.

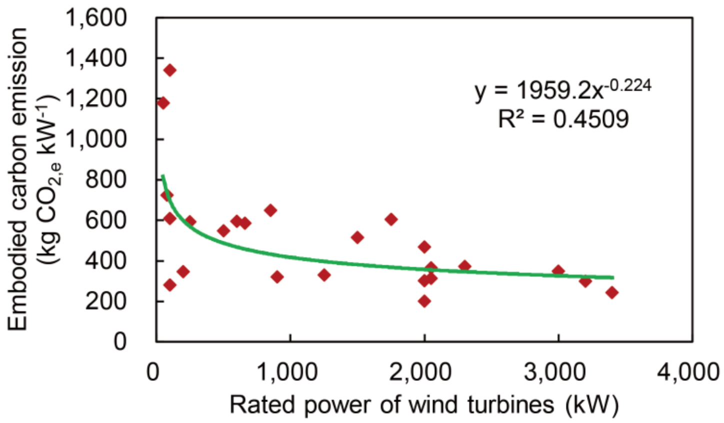

Supplementary Figure 12 The embodied carbon emission of wind turbines. The red dots represent the embodied carbon emissions generated by different models of wind turbines. The green curve represents the fitting curve obtained by exponential fitting of the red dots.

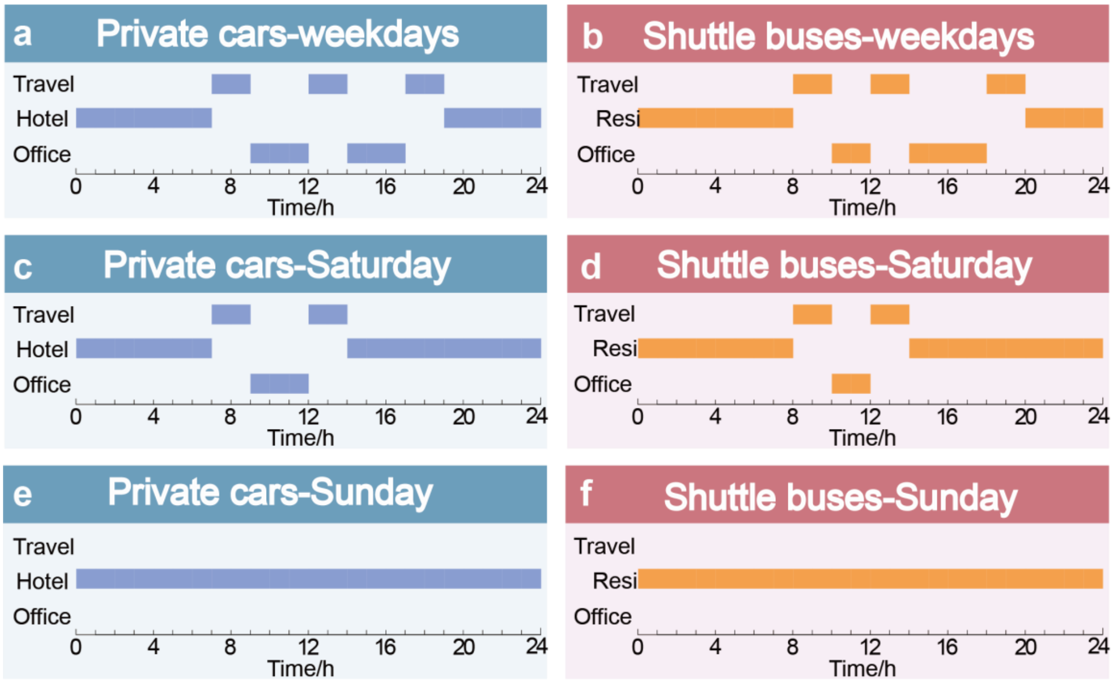

Supplementary Figure 13 The travelling schedule (Schedule B) of private cars. a, b, c The schedule of private cars (Group 1) on weekdays, Saturday and Sunday, respectively; **d, e, f** The schedule of shuttle buses (Group 2) on weekdays, Saturday and Sunday, respectively.

Supplementary Note 1. The methodology description.

The main feature of our method is that it is based on multiple models and is organically combined and used in series based on modular design. As shown in Supplementary Fig. 1, firstly, we obtained a climate database, renewable energy data, vehicle operation data, and electricity price data for different climate zones. Based on these data, considering different energy interaction modes, battery reuse strategies, and the relative relationship between renewable energy supply and energy demand, we reasonably preset 5 scenarios (Scenario A-E) and 3 paradigms (Paradigm A-C). We then established corresponding building-vehicle energy sharing network models and their analytical models, including building thermodynamics models, integrated building energy systems (e.g., building services systems, rooftop PVs, BIPVs, solar thermal collectors, and so on), building-vehicle interaction and battery degradation models. We then analysed the following three projects: life cycle carbon emissions comparison between electric vehicles and internal combustion engine vehicles, calculation of carbon emission factors for renewable energy, and quantifications of battery life cycle carbon intensity, especially considering the complexity in the operational stage. Based on the analysis results, we finally proposed zero-carbon pathways for battery life cycles in different climate zones.

Supplementary Note 2: The operating principle of building-transportation energy sharing network.

The principles of V2B/B2V can be explained by Supplementary Fig. 2. In the building-transportation-storage energy system, renewable energy is divided into a surplus period and a shortage period, according to the relative difference between renewable energy and building energy demand. During the surplus period, excess renewable energy is stored in the battery energy storage system (including EVs) when the energy demand is completely covered. During the shortage period, building energy demands are firstly covered by renewable energy, and then by energy storage systems, before being covered by imported power from the electric grid. Excess renewable energy from the office building will be managed to charge EV group 1 or EV group 2 with the priority given to the EV groups with a lower FSOC. By contrast, the EV group with a higher FSOC will be given the priority for discharging to cover the demand shortage. An off-peak mandatory charging is applied to meet the vehicle's travelling demands during the daytime. For EV group 1 and EV group 2, the maximum charge and discharge power are 10 kW and 20 kW, respectively. In addition, EVs only participate in V2B/B2V when parked next to the corresponding building.

For private cars, their daily travelling time is 6 hours on weekdays, and 4 hours on Saturdays, and does not travel on Sundays (Called "Schedule B"). In order to explore the impact of different scheduling on battery performance and carbon intensity, this study assumes two other schedules, as shown in Supplementary Fig. 3 ("Schedule A" and "Schedule C"). Then, they are compared with the case without multi-directional V2X, in terms of the depth of discharge and degradation degree.

As shown in Supplementary Fig. 4, when there is no multi-directional V2X, the depth of discharge is concentrated in the interval [0.1, 0.2) and [0.2, 0.3). After adding multi-directional V2X, regardless of the schedule of EVs, the distribution of its depth of discharge is more uniform compared to the case without multi-directional V2X, although the maximum distribution still occurs in [0.2, 0.3). In addition, scheduling has less impact on EV battery degradation. As shown in Supplementary Fig. 5, within 20 years of operation, except for the case without multi-directional V2X, all EV batteries need to be replaced three times.

Supplementary Note 3: The charging/discharging cycles, depth of discharge, and degradation of EV batteries in different scenarios.

In Supplementary Figs. 6 and 7, we summarize the charging and discharging cycles, degradation degree, and depth of discharge of EV batteries in different scenarios in Guangzhou. It is worth noting that in scenarios A, B, and D without multi-direction V2X, the number of battery cycles is much less than that in scenarios C and E with multi-direction V2X. In addition, the depth of discharge of scenarios A, B, and D is mainly concentrated at [0.1-0.3), which is the main depth of discharge when the vehicle is travelling, while the DOD distribution of scenarios C and E is more scattered because they begin to store excess renewable energy, and there is uncertainty in the amount of excess renewable energy. Since the degree of battery degradation is affected by a lot of factors, it is difficult to see from Supplementary Figs. 6 and 7 the specific effects of charge/discharge cycle and depth of discharge on battery degradation in different scenarios. Therefore, readers can refer to the battery degradation curve (Supplementary Fig. 8) to see the specific impact. From Supplementary Fig. 8, it's clear that a greater number of cycles will lead to higher battery degradation, and when the depth of discharge is far away from 0.4, the battery degradation will become even more obvious.

As for the battery carbon intensity, neither the charge/discharge cycles nor the depth of discharge has a direct relationship with the carbon intensity. This can be known from the results of Scenarios A, B, and D. Supplementary Table 1 summarizes the relevant results of battery carbon intensity in Guangzhou. It is noted that the carbon intensity varies greatly between different scenarios, with the minimum (Scenario B) at -1,104.91 kg $CO_{2,e}$/kWh and the maximum (Scenario A) at 1,625.01 kg $CO_{2,e}$/kWh. According to Supplementary Figs. 6 and 7, Scenario A and B have the same charge/discharge cycles and depth of discharge, but they have great differences in carbon intensity, which shows that the charge/discharge cycles and depth of discharge are not directly related to carbon intensity.

Supplementary Note 4: Building information and simulation environment.

The E-mobility-based interactive energy-sharing community includes two high-rise buildings (a hotel building and an office building) and 30 single residential buildings modelled by Type 56, TRNBuild[10]. The hotel and office building has 30 floors with an area of 400 m^2 (20 m×20 m), and 625 m^2 (25 m×25 m) for each floor. The residential building has two floors with an area of 300 m^2 (15 m×20 m) for each floor.

Building energy simulation includes two parts: internal gains and HVAC (Heating, ventilation, and air conditioning) system. Internal gains produced by the equipment are 10 W m^{-2}. Internal gains produced by light are 12 W m^{-2} (office building) or 15 W m^{-2} (Hotel and Residential building). Internal gains generated by the human body are 70 W per person. Note that the numbers of people living or working in the hotel, office, and residential building are 16, 40, and 12 respectively.

In the HVAC system part, the cooling system is divided into an air handling unit (AHU) cooling system and a space cooling system, which are used to handle the latent heat and sensible heat respectively. The supply air temperature is 19-23 °C, and the set point temperature is 26 °C. The AHU cooling system includes a heat recovery system with a heat recovery efficiency of 0.75. The heating system includes an air handling unit (AHU) heating system and a space heating system, with a set point temperature of 18 °C. The daily hot water consumption at 60 °C for the hotel, office, and residential building is 2.24 m^3 (floor·day)$^{-1}$, 0.148 m^3 (floor·day)$^{-1}$, 0.736 m^3 (floor·day)$^{-1}$, respectively. To cover the demand for hot water, solar thermal collectors of 200 m^2, 90 $m^{2,}$ and 150 m^2 are installed in the hotel, office, and residential buildings, respectively.

Supplementary Note 5: Meteorological parameters and climate information.

The weather file is provided by Meteonorm 5[11].

In Beijing (39.9 °N, 116.1 °E) as shown in Supplementary Fig. 10a-10b, the monthly average wind speed fluctuates between 1.81 and 3.20 m s^{-1}, the monthly average ambient temperature fluctuates between -4.32 and 25.94 °C, and the monthly solar radiation fluctuates between 54.82 and 155.10 kWh m^{-2}. In Guangzhou (23.1 °N, 113.3 °E) as shown in Supplementary Fig. 10c-10d, the monthly average wind speed fluctuates between 2.10 and 2.60 m s^{-1}, the monthly average ambient temperature fluctuates between 12.77 and 28.56 °C, and the monthly solar radiation fluctuates between 62.67 and 167.85 kWh m^{-2}. In Hong Kong (22.3 °N, 114.2 °E) as shown in Supplementary Fig. 10e-10f, the monthly average wind speed fluctuates between 3.6 and 5.7 m/s, the monthly average ambient temperature fluctuates between 14.27 and 28.81 °C, and the monthly solar radiation fluctuates between 63.58 and 167.81 kWh·m^{-2}. In Kunming (25.0 °N, 102.7 °E) as shown in Supplementary Fig. 10g-10h, the monthly average wind speed fluctuates between 1.70 and 3.00 m/s, the monthly average ambient temperature fluctuates between 7.57 and 19.79 °C, and the monthly solar radiation fluctuates between 81.15 and 203.58 kWh·m^{-2}. In Shanghai (31.2 °N, 121.5 °E) as shown in Supplementary Fig. 10k-10l, the monthly average wind speed fluctuates between 2.69 and 3.30 m s^{-1}, the monthly average ambient temperature fluctuates between 3.83 and 27.74 °C, and the monthly solar radiation fluctuates between 59.31 and 171.69 kWh·m^{-2}. In Shenzhen (22.5 °N, 114.1 °E) as shown in Supplementary Fig. 10k-10l, the monthly average wind speed fluctuates between 3.09 and 4.16 m/s, the monthly average ambient temperature fluctuates between 14.59 and 28.86 °C, and the monthly solar radiation fluctuates between 64.65 and 161.78 kWh·m^{-2}. In Xi’an (34.3 °N, 108.9 °E) as shown in Supplementary Fig. 10m-10n, the monthly average wind speed fluctuates between 1.46 and 2.12 m s^{-1}, the monthly average ambient temperature fluctuates between -0.44 and 26.27 °C, and the monthly solar radiation fluctuates between 60.64 and 219.90 kWh·m^{-2}. In New York (40.4 °N, 74.0 °W) as shown in Supplementary Fig. 10o-10p, the monthly average wind speed fluctuates between 4.52 and 6.19 m s^{-1}, the monthly average ambient temperature fluctuates between -1.73 and 24.71 °C, and the monthly solar radiation fluctuates between 51.02 and 189.32 kWh·m^{-2}. In Berlin (52.5 °N, 13.4 °E) as shown in Supplementary Fig. 10q-10r, the monthly average wind speed fluctuates between 3.58 and 6.15 m s^{-1}, the

monthly average ambient temperature fluctuates between -0.16 and 18.91 °C, and the monthly solar radiation fluctuates between 13.39 and 157.34 kWh·m^{-2}. In Singapore (1.3 °N, 103.9 °E) as shown in Supplementary Fig. 10s-10t, the monthly average wind speed fluctuates between 1.41 and 2.59 m s^{-1}, the monthly average ambient temperature fluctuates between 25.73 and 27.53 °C, and the monthly solar radiation fluctuates between 118.04 and 149.26 kWh·m^{-2}.

Supplementary Note 6: Parameters and configurations of renewable systems.

The building community involved in the study used BIPV and wind turbines as equipment for on-site renewable energy generation. On hotels, office buildings, and residential buildings, the installed BIPV areas are 6,144, 7,680, and 10,752 square meters respectively, with a total installed capacity of 5,003.7 kWp (Note: kWp means kilowatt peak power). The installed PV module is Hiku-CS3W-450MS[1], and the detailed parameters are shown in Supplementary Table 3. The wind power generation equipment used includes three models of 20 kW, 150 kW, and 500 kW, and this equipment is installed with different capacities in different scenarios as shown in Supplementary Tables 4-6. The three installed models are Hummer-H13.2-20 kW[4], AN Bonus 150-150kW[2], and Vestas-V39-500 kW[3], and their parameters and wind speed-power curve are shown in Supplementary Tables 7 and Supplementary Fig. 11.

Supplementary Note 7: The electricity price of grid power in different cities.

This note introduces electricity prices in the studied cities. Supplementary Tables 8-22 give the commercial and residential electricity prices in Hong Kong, Beijing, Guangzhou, Shenzhen, Xi’an, Shanghai, Kunming, and Singapore respectively. The electricity price in New York can be seen from Ref.[12], the electricity price in Berlin is 0.7 US$ kWh^{-1}, the electricity price of residential buildings in Singapore is 0.2325 US$ kWh^{-1}.

Supplementary Note 8: The additional equations for NPV calculation.

$$\Delta C_{\text{imp,save}}=\sum_{n=1}^{20}\frac{\int_0^{8760}[P_{\text{imp,Case 0},n}(t)\cdot C_{\text{eg,imp}}(t)]dt-\int_0^{8760}[P_{\text{imp,Case X},n}(t)\cdot C_{\text{eg,imp}}(t)]dt}{(1+r)^n}(1+\eta)^n \quad (1)$$

$$\Delta C_{\text{recyc}}=C_{\text{recyc,Case X}}-C_{\text{recyc,Case 0}}=\sum_{n=1}^{20}\frac{\sum_{i=1}^{j}C_{\text{recyc,Case X,i},n}-\sum_{i=1}^{j}C_{\text{recyc,Case 0},i,n}}{(1+r)^n} \quad (2)$$

$$\Delta C_{\text{remanu}}=C_{\text{remanu,Case X}}-C_{\text{remanu,Case 0}}=\sum_{n=1}^{20}\frac{\sum_{i=1}^{j}C_{\text{remanu,Case X},i,n}-\sum_{i=1}^{j}C_{\text{remanu,Case 0},i,n}}{(1+r)^n} \quad (3)$$

$$\Delta C_{\text{repl,new}}=\sum_{n=1}^{20}\frac{\sum_{i=1}^{j}C_{\text{repl,new,Case X},i,n}}{(1+r)^n} \quad (4)$$

$$\Delta C_{\text{repur}}=\sum_{n=1}^{20}\frac{\sum_{i=1}^{j}C_{\text{repur,Case X},i,n}}{(1+r)^n} \quad (5)$$

$$\Delta C_{\text{O\&M}}=C_{\text{O\&M,Case X}}-C_{\text{O\&M,Case 0}}=$$
$$\sum_{n=1}^{20}\frac{\sum_{i=1}^{j}C_{\text{O\&M-battery,Case X},i,n}+C_{\text{O\&M-RE,Case X},n}-\sum_{i=1}^{j}C_{\text{O\&M-battery,Case 0},i,n}-C_{\text{O\&M-RE,Case 0},n}}{(1+r)^n} \quad (6)$$

$$\Delta IC_{\text{RE}}=Cap_{\text{BIPV}}\cdot C_{\text{BIPV}}+Cap_{\text{WT}}\cdot C_{\text{WT}} \quad (7)$$

The subscript n refers to the nth year. *i* refers to the *i*th battery and *j* represents the last battery. The variables η and r is the escalation rate of grid electricity and the interest rate with a value of 1.4% and 2%, respectively. The Case 0 and Case X refer to the Scenario A and other Scenarios in this article, respectively. $P_{\text{imp}}(t)$ refers to the grid import power. $C_{\text{eg,imp}}(t)$ refers to the electricity price of the electricity grid. C_{recyc} (57 \$ kWh^{-1}), C_{remanu} (40 \$ kWh^{-1}), C_{repl} (125 \$ kWh^{-1}), and C_{repur} (35 \$ kWh^{-1}) refer to the recycling, replacement, remanufacturing and repurposing cost for EV batteries and secondary-life batteries. $C_{\text{O\&M}}$ refers to the operation and maintenance cost, which is 0.5% for batteries and 5% for renewable systems annually. Cap_{BIPV} and Cap_{WT} are the total installed capacity of BIPVs and wind turbines. C_{BIPV} and C_{WT} refer to the price of BIPV (240 \$ kWp^{-1}) and wind turbines (400 \$ kW^{-1}).

Supplementary Note 9: Parameters, scheduling, and carbon emission results for EVs and ICEVs.

In this study, the interactive energy network includes two vehicle groups, namely Group 1 and Group 2. When vehicle groups are composed of electric vehicles (EVs), Group 1 is mainly composed of Tesla Model Y as shown in Supplementary Table 23, and Group 2 is mainly composed of Yutong E8 as shown in Supplementary Table 23. When vehicle groups are composed of internal combustion engine vehicles (ICEVs), Group 1 is mainly composed of Honda Accord 2023 as shown in Supplementary Table 24, and Group 2 is mainly composed of Yutong C8 as shown in Supplementary Table 24. Supplementary Fig. 13 shows the operation time of electric vehicles.

In addition, Supplementary Table 25 shows the carbon emission data of different vehicles, including body production, battery production, body recycling, and battery recycling. Supplementary Table 26 shows the carbon emission data of vehicles during the operation phase in different cities.

Supplementary References

1. Csisolar. Hiku-CS3W-450MS, https://static.csisolar.com/wp-content/uploads/sites/9/2019/12/07115154/CS-Datasheet-HiKu_CS3W-MS_v5.9_CN.pdf. (2019).

2. Bonus A. AN Bonus 150/30, https://en.wind-turbine-models.com/turbines/124-an-bonus-150-30. (2005).

3. Vesta. Vestas-V39, https://en.wind-turbine-models.com/turbines/383-vestas-v39. (2017).

4. Hummer. Hummer H13.2-20kW, https://en.wind-turbine-models.com/turbines/1688-hummer-h13.2-20kw. (2017).

5. Tesla. Tesla model Y Long Range, https://ev-database.org/car/1619/Tesla-Model-Y-Long-Range-Dual-Motor. (2022).

6. Yutong. Yutong E8, http://www.yutong.com/products/E8i.shtml., (2022).

7. Zhou Y. Low-carbon transition in smart city with sustainable airport energy ecosystems and hydrogen-based renewable-grid-storage-flexibility. *Energy Reviews* **1(1)**, 100001 (2022).

8. Honda. 2023 Honda Accord,

https://www.fueleconomy.gov/feg/Find.do?action=sbs&id=46363. (2023).

9. Yutong. Yutong C8, https://www.yutong.com/products/ZK6827H.shtml., (2022).

10. UW-Madison. SEL (Solar Energy Laboratory, Univ. of Wisconsin-Madison), TRANSSOLAR (TRANSSOLAR Energietechnik GmbH), CSTB (Centre Scientifique et Technique du Bâtiment). "Multizone Building (Type56 – TRNBuild) for the TRNSYS Simulation Environment, Volume 5 Multizone Building modeling with Type56 and TRNBuild.".) (2017).

11. Meteotest. Meteonorm 5, Asia, https://meteonorm.com/. (2003).

12. Wang Y, Song Z, De Angelis V, Srivastava S. Battery life-cycle optimization and runtime control for commercial buildings demand side management: A New York City case study. *Energy* **165**, 782-791 (2018).
